# Increase in activin A may counteract decline in synaptic plasticity with age

**DOI:** 10.3389/fnagi.2024.1382492

**Published:** 2024-04-05

**Authors:** Fang Zheng, Marc Dahlmanns, Philipp Kessler, Christian Alzheimer

**Affiliations:** Institute of Physiology and Pathophysiology, Friedrich-Alexander-Universität Erlangen-Nürnberg, Erlangen, Germany

**Keywords:** activin, age, synaptic plasticity, LTP, hippocampus

## Abstract

Activin A, a member of the transforming growth factor β (TGF-β) family, is widely recognized for its neurotrophic and neuroprotective function in the developing and injured brain, respectively. Moreover, in the healthy adult brain, activin A has been shown to tune signal processing at excitatory synapses in a fashion that improves cognitive performance. Because its level in human cerebrospinal fluid rises with age, we wondered whether activin A has a role in mitigating the gradual cognitive decline that healthy individuals experience in late-life. To interrogate the role of activin A in synaptic plasticity in the aging brain, we used an established transgenic mouse line, in which expression of a dominant-negative mutant of activin receptor IB (dnActRIB) serves to disrupt activin receptor signaling in a forebrain-specific fashion. In brain slices of young adult dnActRIB mice (2–4 months old), the NMDA receptor-dependent and -independent forms of long-term potentiation (LTP) at the Schaffer collateral—CA1 pyramidal cell synapse of the hippocampus were equally impaired relative to the extent of LTP measured in the wild-type preparation. Unexpectedly, the difference between the genotypes disappeared when the two forms of LTP were re-examined in slices from middle-aged mice (13–16 months old). Since the level of activin A and endogenous ActRIB both displayed a significant elevation in middle-aged hippocampus, we reasoned that with such a rise, the dominant-negative effect of the mutant receptors could be overcome. Substantiating this idea, we found that administration of recombinant activin A was indeed capable of restoring full-blown LTP in slices from young dnActRIB mice. Our data suggest that, beginning in the middle-aged brain, endogenous activin receptor signaling appears to become strengthened in an attempt to stave off cognitive decline. If further corroborated, this concept would also hold promise for new therapeutic venues to preserve cognitive functions in the aged brain.

## Introduction

Despite a wealth of human and animal studies at all levels of investigation, the neural mechanisms propelling the gradual cognitive decline in the aging brain of healthy individuals are only partially understood (Burke and Barnes, 2006, 2010; Lister and Barnes, 2009; Small et al., 2011; Gray and Barnes, 2015; Lester et al., 2017; Radulescu et al., 2021). A cognitive function prominently affected in older adults is spatial memory. Orientation in and discrimination of previously visited environments require subfield-specific information processing in different regions of the hippocampus (Lester et al., 2017). Using high-resolution functional MRI, which enables simultaneous subfield imaging, Zheng et al. (2023) showed recently that impaired spatial navigation in older individuals is associated with disturbed information flow toward area CA1 within the hippocampal circuit. The major excitatory input to CA1 pyramidal cells is provided by the Schaffer collaterals (SC), which originate from CA3 pyramidal cells. Long-term potentiation (LTP) of the SC-CA1 pyramidal cell synapse is widely regarded as a neurobiological substrate of a memory trace capable of storing spatial information. Studies in rodent hippocampus on age-related changes in LTP at this synapse yielded inconsistent results, apparently depending on the particular LTP induction protocol and on whether the experiments were performed *in vivo* or in hippocampal slices (Shankar et al., 1998; Barnes, 2003; Rosenzweig and Barnes, 2003; Burke and Barnes, 2006, 2010; Kumar, 2011). When examined in the latter preparation using near-threshold stimulation, the prototypical NMDA receptor-dependent LTP (NMDA-LTP) was found to decline as the animals, from which the slices were obtained, grew old (Moore et al., 1993; Rosenzweig and Barnes, 2003; Burke and Barnes, 2006, 2010).

A possible path to impaired LTP and cognitive decline in older age may be paved by the aging-associated decrease in the level of brain-derived neurotrophic factor (BDNF) in the cerebrospinal fluid (CSF; Li et al., 2009; Erickson et al., 2012). The pleiotropic neurotrophin BDNF is widely recognized as a LTP-promoting factor that is released in an activity-dependent fashion, exerting a number of local effects that all serve to consolidate synaptic plasticity (Korte et al., 1995; Patterson et al., 1996; Leal et al., 2017; Brigadski and Lessmann, 2020). The notion that a lower BDNF level in the aging hippocampus contributes to deterioration of cognitive functions (Silhol et al., 2005; Erickson et al., 2012) receives support from the finding that in brain slices from middle-aged rats, impaired LTP can be restored by exogenously supplied BDNF (Rex et al., 2006). Vice versa, one might expect that a LTP-fostering factor, which exhibits increasing levels with age, should be capable of counteracting impaired plasticity. We posit here that activin A, a member of the transforming growth factor β (TGF-β) family, might assume such a role in the aging hippocampus. In fact, activin A levels in serum and CSF rise with age in individuals of both sexes (Harada et al., 1996; Loria et al., 1998; Baccarelli et al., 2001; Ebert et al., 2006; Baird et al., 2012). In functional terms, activin A shares with BDNF a broad spectrum of neurotrophic and neuroprotective effects in the developing and adult brain, which includes augmentation of hippocampal LTP (Müller et al., 2006; Ageta et al., 2010). As predicted from its role in LTP, activin A makes an impact on the performance of rodents in learning and memory tasks (Ageta et al., 2010). Moreover, activin A also regulates anxiety- and depression-like behavior, which has been linked in part to the modulation of GABAergic synapses (Zheng et al., 2009; Ageta and Tsuchida, 2011; Krieglstein et al., 2011; Link et al., 2016b; Mitra et al., 2022).

Activin A, homodimer of two disulfide-linked βA subunits, exerts its effects via binding to heterotetrameric receptor complexes containing two type II activin receptors and two type I activin receptors (mainly activin receptor type IB, ActRIB). Activated receptors phosphorylate the intracellular signaling proteins SMAD2/3, which co-assemble with SMAD4 and translocate to the nucleus to regulate gene transcription. In addition to the canonical SMAD2/3 pathway, other pathways are also activated by activin receptors, including MAPK- as well as PKA- and PKC-mediated signaling (Tsuchida et al., 2009; Bloise et al., 2019). Whereas activin A levels in the mature brain are low under resting conditions, they show a massive, but transient surge after brief trains of LTP-inducing stimuli (Andreasson and Worley, 1995; Inokuchi et al., 1996) and after behavioral stimulation such as provided by environmental enrichment (EE; Link et al., 2016a; Jaeger et al., 2018; Dahlmanns et al., 2023a). Lending credence to the functional significance of the strong rise in endogenous activin A in the EE paradigm, we found that its effects on CA1 pyramidal cell excitability, neurotransmission, and synaptic plasticity were largely abrogated in transgenic mice expressing a dominant-negative mutant of activin receptor IB (dnActRIB; Dahlmanns et al., 2023a). In this mouse model of disrupted activin receptor signaling, the transgene is expressed under the control of the CaMKIIα promoter, ensuring that its expression is restricted to forebrain neurons and only begins when the early postnatal phase is over (Müller et al., 2006). In the present study, we report that compared to hippocampi from young mice, those from older mice exhibit higher levels of both activin A and activin receptor IB. We explore how this elevation affects the neurophysiological properties of the SC-CA1 pyramidal cell synapse in hippocampi from wild-type and dnActRIB mice of young and old age.

## Materials and methods

### Animals

Our experiments were performed on hippocampi prepared from adult male and female mice of young (2–4 months; i.e., young adults) and middle-aged (13–16 months; i.e. old adults) mice, which were either wild-type (wt, *n* = 34 young and *n* = 29 middle-aged) with C57Bl/6 J background or littermate transgenic mice expressing a dominant-negative mutant of ActRIB (dnActRIB, *n* = 37 young and *n* = 29 middle-aged) (Müller et al., 2006). Mice were housed under standard conditions. All experiments were conducted in accordance with the Animal Protection Law of Germany and the European Communities Council Directive of November (1986 /86/609/EEC) and were approved by local government of Lower Franconia.

### Hippocampal slice preparation and electrophysiological recordings

Transverse or horizontal slices (350 μm thick) from dorsal hippocampus were prepared from mice anesthetized with isoflurane, as described previously (Müller et al., 2006; Dahlmanns et al., 2023a,b). Briefly, brain slices were cut in ice-cold sucrose-based artificial cerebrospinal fluid (aCSF) containing (in mM) 75 sucrose, 87 NaCl, 3 KCl, 0.5 CaCl_2_, 7 MgCl_2_, 1.25 NaH_2_PO_4_, 25 NaHCO_3_, and 10 d-glucose. Slices were incubated in the same solution for 10 min at 35°C and then maintained in aCSF containing (in mM) 125 NaCl, 3 KCl, 1 CaCl_2_, 3 MgCl_2_, 1.25 NaH_2_PO_4_, 25 NaHCO_3_, and 10 d-glucose at room temperature for at least 2 h before being used. Individual slices were transferred to a submerged chamber that was mounted on the stage of an upright microscope, and perfused with normal aCSF with 1.5 mM MgCl_2_ and 2.5 mM CaCl_2_ at 31°C. All solutions were constantly gassed with 95% O_2_ / 5% CO_2_. Recorded signals were filtered at 2 kHz and sampled at 20 kHz using a Multiclamp 700B amplifier together with Digidata 1440A interface and pClamp10 software (Molecular Devices, Sunnyvale, CA, United States).

Field potentials in CA1 *stratum radiatum* were recorded with a glass pipette filled with modified aCSF, in which NaHCO_3_ was replaced by HEPES (5 mM) to avoid pH change. A concentric platinum bipolar electrode was inserted into the *stratum radium* to stimulate Schaffer collaterals (SC), with constant current pulses (0.1 ms width). The input–output relationship of the SC-CA1 synapse was routinely determined by an incremental increase in stimulus strength from 50 to 200 μA. Short-term potentiation (STP) and long-term potentiation (LTP) of the SC-CA1 synapse were examined using stimulus intensities that elicited 30% of the maximum response before high frequency stimulation. With individually adjusted stimuli delivered at 0.1 Hz before and after tetanus, two forms of SC-CA1 LTP were studied. “Classic” NMDA receptor-dependent LTP was induced by applying theta-burst stimuli (TBS: 15 bursts of four pulses at 100 Hz, delivered at an inter-burst interval of 200 ms), twice 10 s apart. Voltage-gated Ca^2+^ channel-dependent LTP was measured in the presence of the NMDA receptor blocker APV (50 μM) and induced by strong high-frequency stimulation at 200 Hz (for 1 s), repeated four times at 5 s intervals (Grover and Teyler, 1990). Data points were obtained by averaging six responses every minute.

### Enzyme-linked immunosorbent assay

Mice were sacrificed under anesthesia with isoflurane and the brain was quickly dissected out. The isolated hippocampus was homogenized in lysis buffer containing 0.32 M sucrose, 5 mM Tris–HCl (pH 8.0), and a protease inhibitor cocktail (Sigma-Aldrich, St. Louis, MO, United States). Homogenates were centrifuged at 13,000 × *g* at 4°C for 15 min (twice). Supernatant was collected for assaying levels of activin A and ActRIB according to the manufacturer’s instructions, with ELISA kits from R&D Systems (Activin A Quantikine ELISA Kit; Minneapolis, MN, United States) and ELAab Science Co. Ltd. (Activin Receptor Type IB ELISA Kit; Wuhan, China), respectively.

### Reverse transcription quantitative real-time PCR

Brains from young and old adult wt and dnActRIB mice were extracted as described above, and the hippocampi were isolated and stored at −80°C. RNA was then isolated according to the manufacturer’s protocol (RNeasy Plus Universal Mini Kit; QIAGEN, Hilden, Germany), reverse-transcribed into cDNA (High-Capacity cDNA Reverse Transcription Kit; Applied Biosystems, Waltham, MA, United States), and then stored at −20°C. RNA levels were determined using quantitative real-time PCRs (RT-qPCR) according to the manufacturer’s instructions (ABsolute QPCR Mix SYBR Green no ROX, Thermo Fisher Scientific, Waltham, MA, United States) in a realple × 4 cycler (Eppendorf, Hamburg, Germany). The following primers were used (Eurofins Genomics, Ebersberg, Germany): (1) dnActRIB (NM_007395.3, exon 6–7 and exon 7–8 deleted in the dnActRIB mutant, 130 bp), 5′-tctaccataaccgccagagg-3′ (forward) and 5′-aggtcctcctcggaaatcag-3′ (reverse), and (2) TATA binding protein (TBP; 146 bp), 5′-gccaagagtgaagaacaatcc-3′ (forward) and 5′-ccttccagccttatagggaac-3′ (reverse). Every biological sample was measured as a technical duplicate and then averaged. Then, ΔCq values were calculated by relating dnActRIB to TBP, and 2^−ΔΔCq^ values were calculated by normalizing values to the mRNA abundance of ActRIB in the hippocampus of dnActRIB mice.

### Data analysis and statistics

Electrophysiological data analysis was performed off-line with Clampfit 10.2 (Molecular Devices, Sunnyvale, CA, United States). Peak amplitude and initial slope (20–80% of the peak response) of field postsynaptic potentials (fPSPs) were quantified. STP during quadruple-pulse stimulation was determined by normalization peak amplitude of 2nd–4th response to that of first response. For LTP measurements, averaged fPSP slopes (from six responses every minute) were normalized to 100% before tetanus and pooled across mice of the same group.

Data are expressed as means ± SEM. OriginPro 2018G (OriginLab Corporation, Northampton, MA, United States) was used for statistics and graphs. Shapiro–Wilk test was used to assess the normality of data distribution, and the null hypothesis was accepted when *p* value was larger than 0.05. Statistical comparisons were performed using unpaired or paired Student’s *t*-test, one-way ANOVA followed by Tukey’s *post-hoc* test, as appropriate. Significance was assumed for *p* < 0.05.

## Results

### In aging hippocampus, expression of dominant-negative activin receptors loses influence on plasticity of Schaffer collateral-CA1 synapse

**Figure 1:**
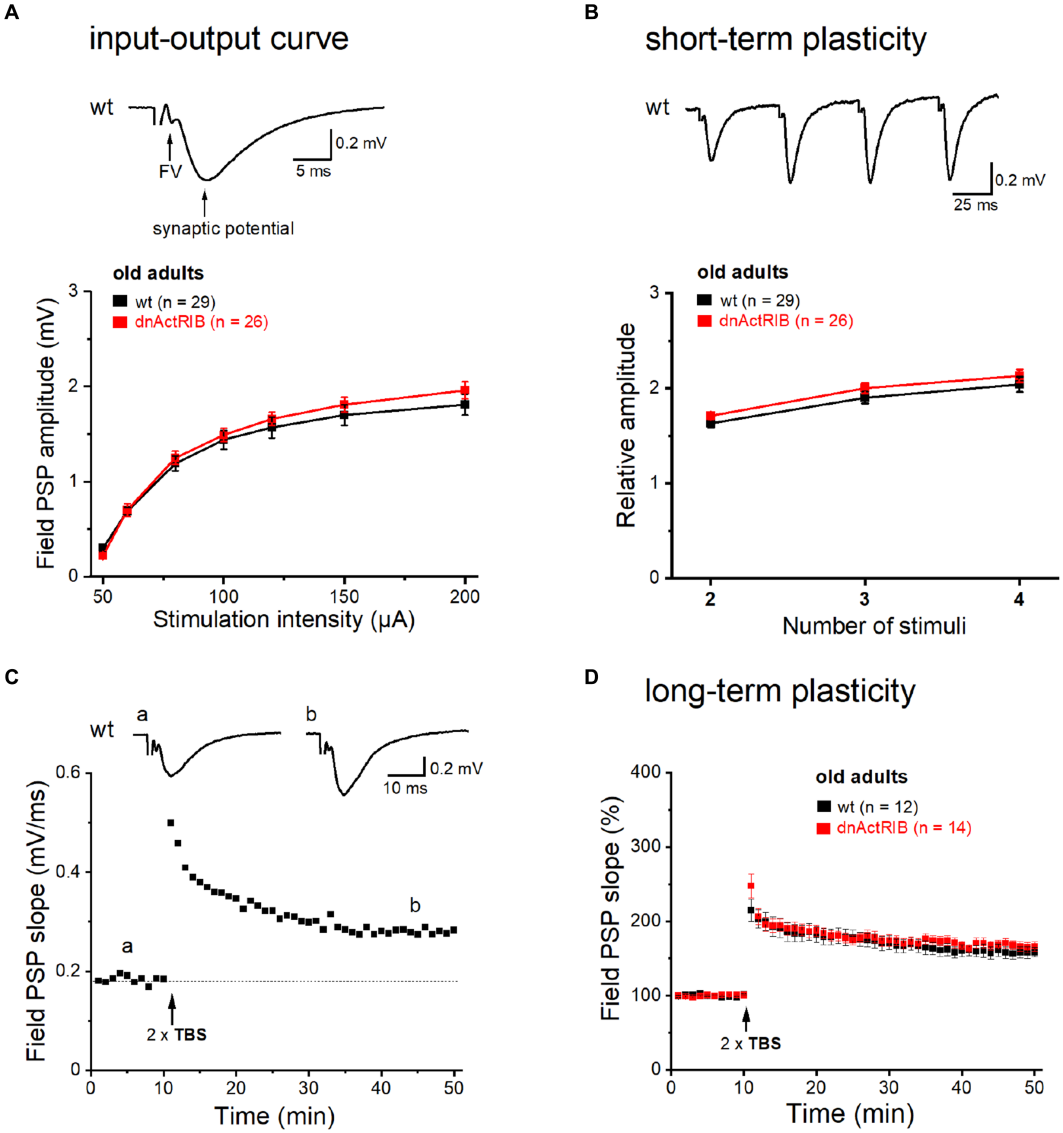
Basic neurotransmission and synaptic plasticity in area CA1 are unchanged in aged dnActRIB mice. Field potentials were evoked and monitored by means of extracellular electrodes placed in CA1 stratum radiatum. **(A)** I-O curves for fPSPs at the SC-CA1 synapse were constructed for wt (black) and mutant preparations (red). Representative voltage trace above I-O curves was recorded in slice from 15 months old wt mouse. Electrical stimulation (50 μA) of Schaffer collaterals evoked a sequence of field potentials consisting of axonal response (fiber volley, FV) and subsequent postsynaptic response (fPSP). Stimulus artifact was truncated. **(B)** Short-term plasticity was probed by quadruple-pulse stimulation. The increase in 2nd to 4th fPSP relative to 1st one did not differ between genotypes. Representative voltage trace above curves illustrates characteristic potentiation of fPSPs by the four stimuli (20 Hz, 60 μA) in slice from 16 months old wt mouse. **(C,D)** TBS-induced NMDA-LTP of SC-CA1 synapse did not differ between genotypes (**D**). Time course of fPSPs potentiation in response to TBS stimulation in wt slice from representative experiment is depicted in panel **(C)**. Insets show averaged voltage traces before (a) and 36–40 min (b) after TBS.

We reported previously that in the hippocampus from young mice expressing a dominant-negative mutant of ActRIB (dnActRIB), several features of basal excitatory neurotransmission and synaptic plasticity were altered when compared to wt mice (Müller et al., 2006; Dahlmanns et al., 2023a,b). To examine how age alone and in combination with activin receptor signaling would affect the performance of glutamatergic synapses, we made extracellular recordings in the CA1 region of brain slices from middle-aged wt and dnActRIB mice. Electrodes placed in *stratum radiatum* served to measure field postsynaptic potentials (fPSPs) in response to electrical stimulation of Schaffer collaterals (SC; Figure 1A, *inset*). As a basic property, we determined first the input–output (I-O) relationship of the SC-CA1 synapse, which depicts peak fPSP amplitude as function of stimulus intensity (50–200 μA, Figure 1A). Whereas the I-O relationship of the SC-CA1 synapse differed significantly between wt and mutant mice of young age, as reported before (Dahlmanns et al., 2023a,b), the I-O curves in hippocampi from older wt and dnActRIB mice displayed a nearly perfect overlap, with indistinguishable responses across stimulating intensities (e.g., at 200 μA: wt-old, 1.81 ± 0.11 mV, *n* = 29; dnActRIB-old, 1.96 ± 0.09 mV, *n* = 26; *p* = 0.404, unpaired *t*-test; Figure 1A). We next asked whether the characteristic facilitation of synaptic responses upon repetitive stimulation would be impaired in the CA1 region of older dnActRIB mice. We gauged frequency facilitation by means of a train of four identical stimuli delivered at 20 Hz (Figure 1B). For comparison, fPSP amplitudes were normalized to that of the first response. Again, no difference was observed between hippocampi from the two groups of older mice (ratio for the 4th fPSP: wt-old, 2.04 ± 0.08, *n* = 29; dnActRIB-old, 2.13 ± 0.07, *n* = 26; *p* = 0.345, unpaired *t*-test; Figure 1B).

Using weak to moderate theta-burst stimulation (TBS) protocols, we found previously that activin signaling augments NMDA receptor-dependent LTP at the SC-CA1 synapse in young adult mice (Müller et al., 2006; Dahlmanns et al., 2023a,b). Here, we repeated this experiment in slices from older wt mice. Employing a comparably moderate TBS paradigm (2 × TBS, separated by 10 s), we observed an increase in fPSP slope to 157.73 ± 7.22% of control value when analyzed 36–40 min after TBS (*n* = 12; Figures 1C,D). Notably, the amount of potentiation was not significantly different from that previously determined in young wt hippocampus (Müller et al., 2006). However, in contrast to the plastic deficit in the young mutant, time course and extent of TBS-induced LTP were virtually identical in hippocampi from both genotypes when measured in the older cohorts (dnActRIB-old, 166.78 ± 6.02%, *n* = 14; *p* = 0.342 vs. wt-old, unpaired *t*-test; Figure 1D).

**Figure 2:**
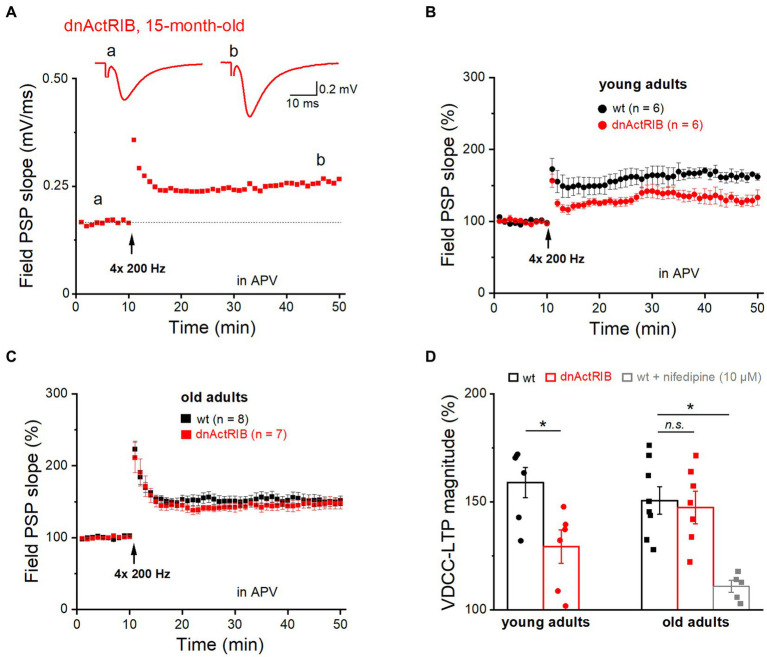
Mutant activin receptor impairs VDCC-LTP in young, but not in old hippocampus. VDCC-LTP was induced by four blocks of high-frequency stimulation at 200 Hz (for 1 s), in the presence of APV (50 μM). **(A)** Trajectory of fPSP slope before and after the heavy stimulation required to elicit VDCC-LTP in slice from 15 months old dnActRIB mouse. Traces above depict averaged fPSPs during baseline (a) and 36–40 min after tetanus (b). Dashed line indicates the averaged value of field PSP slope before tetanus. **(B,C)** Impaired VDCC-LTP in mutant (red) compared to wt hippocampus (black) was observed only in slices from young mice **(B)**, but not from old mice **(C)**. **(D)** Histogram summarizes strength of VDCC-LTP at 36–40 min post tetanus in young and old specimens of both genotypes. Suppression of VDCC-LTP in the presence of L-type Ca^2+^ channel blocker nifedipine (10 μM). Statistical comparisons were performed using an unpaired, two-tailed student’s *t*-test at *α* = 0.05 (**D**, young adults) or a one-way ANOVA followed by Tukey’s *post-hoc* test (**D**, old adults, *F* = 8.995, *p* = 0.009). *n.s.*, not significant; ^*^p < 0.05.

In addition to “classic” NMDA receptor-mediated LTP, the SC-CA1 synapse is known to exhibit an NMDA receptor-independent form of LTP that requires much stronger stimulation (200 Hz trains for 1 s, repeated four times every 5 s), and relies on activation of voltage-gated Ca^2+^ channels (VDCC; Grover and Teyler, 1990). Notably, the latter form of LTP (VDCC-LTP) was found to increase in aged rats (Shankar et al., 1998; Boric et al., 2008), as does the L-type Ca^2+^ current in CA1 pyramidal cells of old rats (Thibault and Landfield, 1996). Whether the increase in this type of LTP can be regarded as a compensatory mechanism for the aging-associated decline in NMDA receptor-dependent LTP to preserve cognitive function in old rodents remains unclear (Burke and Barnes, 2010; Foster, 2012). To examine whether activin signaling is also involved in VDCC-LTP, we subjected hippocampi from younger and older mice of both genotypes to the strong induction protocol described above (Figure 2). This set of experiments, which we performed in the presence of the NMDA receptor antagonist APV (50 μM), yielded the following results: (i) The magnitude of VDCC-LTP in hippocampi from young dnActRIB mice was significantly smaller compared to that from young wt mice (36–40 min post-tetanus: wt-young: 158.98 ± 7.00%, *n* = 6; dnActRIB-young: 129.31 ± 7.71%, *n* = 6; *p* = 0.017, unpaired *t*-test; Figures 2B,D); (ii) Reminiscent of our finding for NMDA receptor-dependent LTP in older mice (Figure 1D), the remarkable discrepancy in the amount of VDCC-LTP between wt and mutant preparations at young age vanished when re-examined in the older cohorts, which exhibited virtually identical amounts of VDCC-LTP (36–40 min post-tetanus: wt-old: 150.66 ± 6.44%, *n* = 8; dnActRIB-old: 147.39 ± 7.58%, *n* = 7; *p* = 0.741, ANOVA with Tukey’s *post-hoc* test) (Figures 2A,C,D); (iii) There was no difference in VDCC-LTP between young and old control groups (wt-young vs. wt-old, *p* = 0.404, unpaired *t*-test). To verify the Ca^2+^ channel-dependence of this form of LTP, we superfused slices from old adult wt mice with the L-type blocker nifedipine (10 μM). The drug produced a uniform increase in synaptic transmission, which reached steady-state within 16–20 min (from 0.49 ± 0.05 to 0.69 ± 0.06 mV, *n* = 6, *p* = 0.009, paired *t*-test). Reassuringly, nifedipine suppressed the subsequent induction of VDCC-LTP (110.94 ± 2.73% of control value, *n* = 5; *p* = 0.029 vs. wt-old, ANOVA with Tukey’s *post-hoc* test; Figure 2D), confirming the essential involvement of L-type Ca^2+^ channels.

### Aged mice have lighter hippocampus, but higher level of activin A and ActRIB

**Figure 3:**
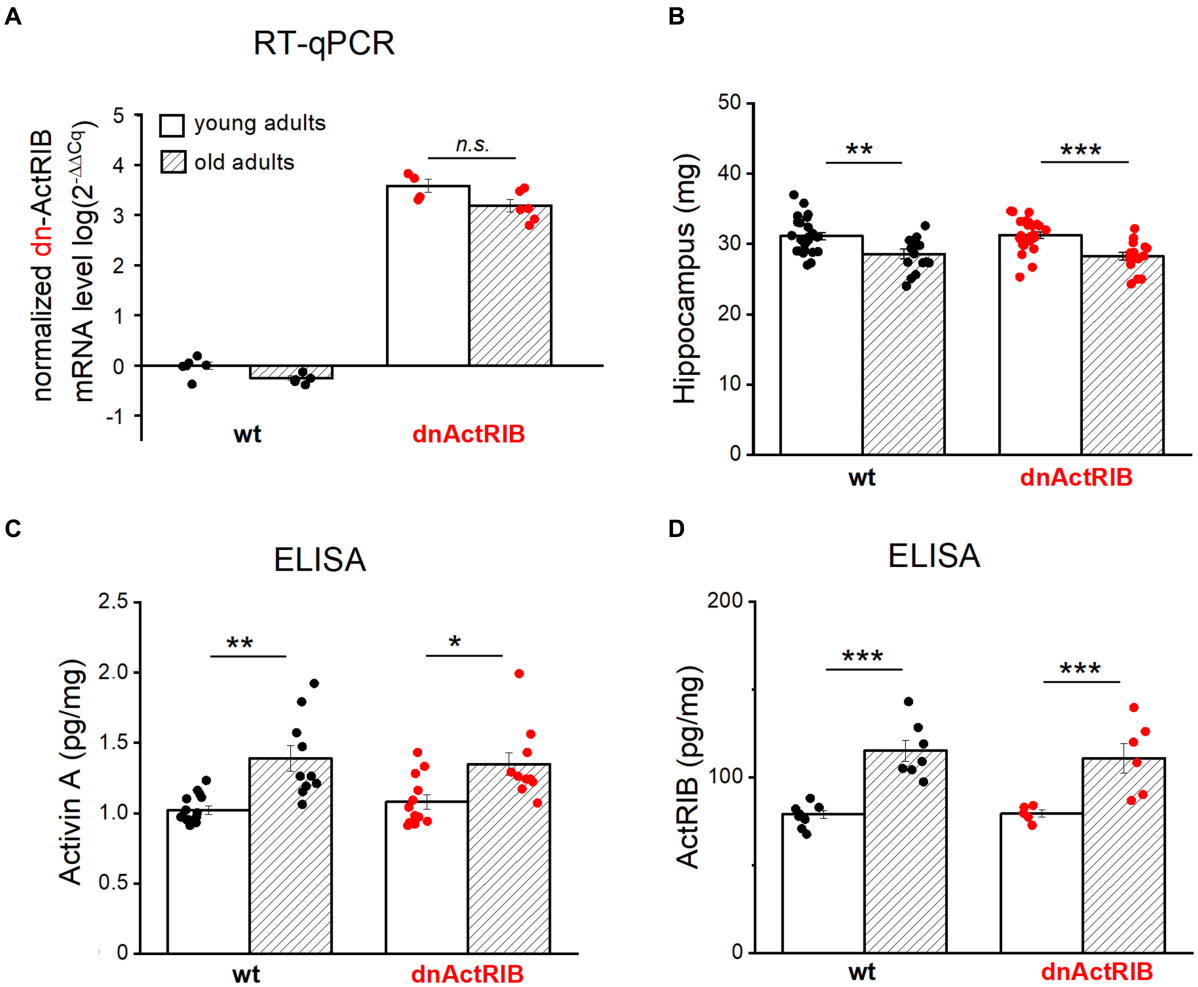
Both activin A and activin receptor IB are increased in aged hippocampus. **(A)** RT-qPCR analysis reveals comparable levels of dnActRIB mRNA in hippocampi of young and old mutant mice. **(B)** Aged mice have lighter hippocampus, compared to young mice. **(C,D)** ELISA measurement shows higher levels of endogenous activin A **(C)** and endogenous activin receptor ActRIB **(D)** in aged hippocampi. Statistical comparisons were performed using an unpaired, two-tailed student’s *t*-test at *α* = 0.05. n.s., not significant, ^*^p < 0.05; ^**^p < 0.01; ^***^p < 0.001.

To substantiate the physiological significance of our findings, we had to rule out that it is simply a decline in transgene expression with age that explains the apparent failure of the dominant-negative receptor approach to replicate the LTP-phenotype of the young mice in the old ones. As an antibody specifically recognizing dominant-negative ActRIB (dn-ActRIB) is not available, we performed RT-qPCR to demonstrate the sustained mRNA abundance of mutant receptors in aged hippocampi (2^-ΔΔCt^ for dnActRIB transgene/β-Actin: dnActRIB-old, 3.19 ± 0.12, *n* = 6), which was not different from that in mutant young adults (dnActRIB-young, 3.58 ± 0.13, *n* = 4; *p* = 0.063, unpaired *t*-test; Figure 3A). As expected, dnActRIB transcripts were absent in wt hippocampi (wt-old, *n* = 6; wt-young, *n* = 5; Figure 3A).

In the human cerebrospinal fluid, the level of activin A increases with age (Ebert et al., 2006; Baird et al., 2012). To determine whether such an age-dependent elevation also holds for mouse hippocampus, we measured the level of activin A in young and aged hippocampi by means of ELISA. When the hippocampi from both hemispheres were prepared for ELISA measurements, we noted a small, but, significant difference in weight between young and old hippocampi (Figure 3B). In quantitative terms, hippocampi from aged wt mice had less weight (wt-old, 28.59 ± 0.66 mg, *n* = 18) compared to those from young wt mice (wt-young, 31.14 ± 0.53 mg, *n* = 24; *p* = 0.004, unpaired *t*-test) (Figure 3B). Such decrease in weight was also true for dnActRIB mice: older hippocampi were lighter (28.26 ± 0.53 mg, *n* = 16) than young ones (31.27 ± 0.44 mg, *n* = 26, *p* = 0.0001, unpaired *t*-test; Figure 3B). ELISA measurements revealed significantly elevated levels of activin A in aged hippocampi of mice with either genotype (wt-old, 1.39 ± 0.09 pg/mg, *n* = 10; wt-young, 1.02 ± 0.03 pg/mg, *n* = 15, *p* = 0.003, unpaired *t*-test; dnActRIB-old, 1.35 ± 0.08 pg/mg, *n* = 10; dnActRIB-young, 1.08 ± 0.05 pg/mg, *n* = 12, *p* = 0.011, unpaired *t*-test; Figure 3C). Importantly, the rise in activin A level with age was accompanied by a parallel increase in the amounts of ActRIB in aged hippocampi from mice of both genotypes (Figure 3D). The comparable levels of functional (endogenous) ActRIB between genotypes in each age group, young hippocampi (wt-young, 78.94 ± 2.34 pg/mg, *n* = 8; dnActRIB-young, 79.34 ± 2.03 pg/mg, *n* = 5, *p* = 0.168, unpaired *t*-test) and old hippocampi (wt-old, 115.20 ± 6.06 pg/mg, *n* = 7; dnActRIB-old, 110.87 ± 8.50 pg/mg, *n* = 6, *p* = 0.750, unpaired *t*-test), argue against the antibody recognizing the dominant negative activin receptors.

### Recombinant activin A restores impaired synaptic plasticity in hippocampus of young dnActRIB mice

**Figure 4:**
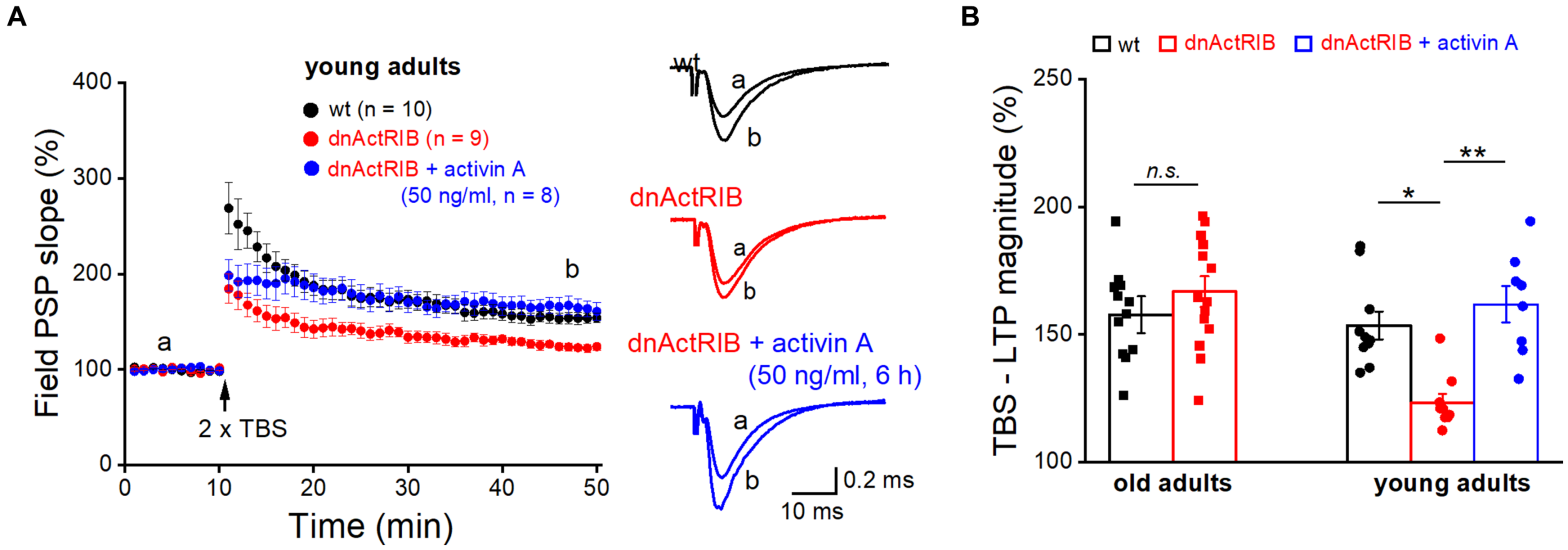
Application of recombinant activin A restores NMDA-LTP in young dnActRIB mice. **(A)** Impaired TBS-induced NMDA-LTP in young dnActRIB hippocampi (red) was rescued after pre-incubation with recombinant activin A (50 ng/mL for 3–8 h, blue), now matching the potentiation seen in wt slices (black). Superimposed traces on the right illustrate extent of fPSP potentiation in each group. **(B)** Histogram summarizes LTP magnitudes measured 36–40 min post TBS in young and old hippocampi of either genotype. Statistical comparisons were performed using an unpaired, two-tailed student’s *t*-test at *α* = 0.05 (**B**, old adults) or a one-way ANOVA followed by Tukey’s *post-hoc* test (**B**, young adults, *F* = 10.071, *p* = 0.002). *n.s.*, not significant; ^*^p < 0.05.

It is tempting to speculate that the age-associated increase in endogenous activin A together with the rise in its main signal-transducing receptor is sufficient to override the inhibitory effect of the dominant-negative receptors on LTP that we observed in young hippocampus where the level of activin A is lower. Thus, we predicted that application of recombinant activin A should restore the extent of LTP in hippocampi from young dnActRIB mice, by enhancing the probability for formation of heterotetramers consisting of functional full-length (endogenous) receptors. As shown in Figure 4A, pre-incubation of slices from young dnActRIB mice with recombinant activin A was indeed capable of rescuing the LTP phenotype. Consistent with findings from our earlier multi-electrode array-based study (Müller et al., 2006), moderate TBS (2 × TBS) of the SC-CA1 synapse in young hippocampi induced significantly weaker LTP in dnActRIB slices compared to wt slices (wt-young, 153.54 ± 5.43%, *n* = 10; dnActRIB-young, 123.16 ± 3.58%, *n* = 9; *p* = 0.018, ANOVA with Tukey’s *post-hoc* test; Figure 4). However, when we employed the same TBS protocol in dnActRIB slices after 3–8 h of incubation with recombinant activin A (50 ng/mL; R&D Systems), the LTP deficit in the mutant hippocampus was fully rescued (Figure 4). The slope of fPSPs was 161.87 ± 7.14% of control value (36–40 min after TBS, dnActRIB-young + activin A, *n* = 8), which was significantly different from that in mutant controls (*p* = 0.002 vs. dnActRIB-young), but not that in wt young controls (*p* = 0.451 vs. wt-young; one-way ANOVA followed by Tukey’s *post-hoc* test).

## Discussion

We report here that in the hippocampus of young adult mice, unconventional VDCC-LTP at the SC-CA1 synapse is reduced when measured under conditions where activin receptor signaling is genetically disrupted by means of a dominant-negative receptor approach. This finding lends further support to the hypothesis that activin receptor signaling is required to obtain full-blown LTP at this synapse. This concept was initially based on recordings of TBS-induced NMDA-LTP in the CA1 region. We explained the diminished NMDA-LTP in the mutant (dnActRIB) hippocampus by the concomitant decrease of NMDA current in CA1 pyramidal cells (Müller et al., 2006). Since VDCC-LTP occurs independent of NMDA receptor activation, activin signaling should affect other targets to augment this form of LTP. Likely candidates are the Ca^2+^ channels themselves, as suggested by earlier cell line studies on their regulation by activin A (Mogami et al., 1995; Fukuhara et al., 1997), and the local handling of cytosolic Ca^2+^ in neuronal compartments, which is also modulated by activin A (Dahlmanns et al., 2023a).

Using moderate (2 × TBS) to strong (4 × 200 Hz) induction protocols, we did not observe appreciable differences in NMDA-LTP or VDCC-LTP between hippocampi from younger and older mice. The lacking effect of aging is in agreement with most studies in aged rodent hippocampus using supra-threshold stimulation (Rosenzweig and Barnes, 2003; Burke and Barnes, 2006, 2010; Ferando et al., 2016). It is only when near-threshold stimulation was employed that a deficit in CA1 LTP emerged in old animals (Moore et al., 1993; Burke and Barnes, 2006, 2010).

To our surprise, both forms of LTP became apparently independent from activin signaling when examined in the aged preparation. In fact, the trajectories of NMDA-LTP and VDCC-LTP exhibited an almost perfect overlap in aged wt and mutant hippocampus. Two points, however, made us reconsider the idea that LTP in aged hippocampus and activin signaling are uncoupled processes: (i) Compared to their younger counterparts, the older hippocampi were endowed with higher levels of activin A and ActRIB, irrespective of genotype; (ii) The dominant-negative receptor approach differs from receptor knockout in that the intact endogenous receptor is still expressed. Typically, the strong surplus of mutant receptors precludes functionally significant activation of the endogenous receptor. At high levels of activin A, however, the probability for formation of functional receptor complexes consisting of the endogenous receptors increases, and activin signaling would be reconstituted. If this hypothesis is correct, application of a high concentration of recombinant activin A should override the suppression of activin receptor signaling and restore full LTP in the hippocampus of young dnActRIB mice. We could indeed verify this prediction experimentally. Thus, it seems conceivable that the age-associated rise in activin A and ActRIB serves to maintain LTP at a stable level, even in aging hippocampus.

There are some caveats to this conclusion, though. For example, we cannot exclude that the increase in endogenous ActRIB in the aged hippocampus occurs predominantly in glial or other non-neuronal cells. Moreover, the rise could be compensatory in nature, possibly reflecting a decline in pathways downstream of the receptor. One might also wonder, whether the relative moderate, age-associated rise in activin A by about 30% is sufficient to overcome the dominant-negative receptors, which are expressed at a constant high level in the aged hippocampus. We should take into account, however, that the ELISA measurements reflect the basal level of endogenous activin A, which is notoriously low. With high frequent stimulation, however, a massive rise in activin βA mRNA ensues (Link et al., 2016a). Thus, even a relative moderate elevation of its basal level might translate into an over-proportional rise in stimulus-induced activin A concentration. As final proof of our hypothesis, one would have to selectively absorb the surge of activin A molecules elicited by the LTP induction protocol, but such an experiment is not feasible in our setting. Nevertheless, our study should provide considerable evidence that activin signaling in the aging brain deserves further examination as a promising target to maintain mental faculties in late-life.

## Data availability statement

The raw data supporting the conclusions of this article will be made available by the authors, without undue reservation.

## Ethics statement

The animal study was approved by local government of Lower Franconia, Bavaria, Germany. The study was conducted in accordance with the local legislation and institutional requirements.

## Author contributions

FZ: Conceptualization, Data curation, Investigation, Methodology, Supervision, Writing – original draft, Writing – review & editing. MD: Data curation, Formal Analysis, Investigation, Methodology, Writing – original draft. PK: Data curation, Investigation, Methodology, Writing – original draft. CA: Conceptualization, Funding acquisition, Project administration, Resources, Supervision, Writing – original draft, Writing – review & editing.
